# Benign metastasizing leiomyoma of the lung

**DOI:** 10.1186/1477-7819-11-279

**Published:** 2013-10-17

**Authors:** Eun Young Ki, Seon Jeong Hwang, Keun Ho Lee, Jong Sup Park, Soo Young Hur

**Affiliations:** 1 Department of Obstetrics and Gynecology, Seoul St. Mary’s Hospital, The Catholic University of Korea, #505 Banpo-Dong, Seocho-Gu, Seoul, South Korea

**Keywords:** Benign metastasizing leiomyoma, Lung, Uterine leiomyoma

## Abstract

Benign leiomyomas of the uterus are uncommonly found in association with benign smooth muscle tumors beyond the confines of the uterus. Benign metastasizing leiomyoma (BML) is a rare disease in which the lung is described to be the most afflicted extrauterine organ. We present a brief review of the literature, along with case reports for four patients who were followed up after resection of a pulmonary lesion or after pathological confirmation by biopsy. The clinical course of BML varies from chronic asymptomatic appearance to rapid progression, leading to respiratory failure and death. Our BML patients did not complain of pulmonary symptoms, such as cough, dyspnea, or chest tightness. Pathology revealed benign leiomyomas with no atypia and mitotic activity <5 per 10 high-power field. Immunohistochemical staining was positive for actin and desmin. A standard treatment for BML has not yet been established. Because of the hormone-sensitive characteristics of BML, treatments are based on hormonal manipulation along with either surgical or medical oophorectomy. Benign metastasizing leiomyoma can be observed in postmenopausal women. We observed four patients who did not receive adjuvant hormonal therapy because they were postmenopausal or perimenopausal. All patients are still healthy and show no evidence of recurrence or progression of the disease.

## Background

Uterine leiomyomas, the most common gynecological neoplasms in women of reproductive age, have a prevalence of about 50% in women older than 30 years and result from clonal proliferation of uterine smooth muscle tissue [1,2]. Very rarely, benign uterine leiomyomas display bizarre growth patterns, including intravascular leiomyomatosis, disseminated peritoneal leiomyomatosis, and benign metastasizing leiomyoma (BML) [3]. Benign metastasizing leiomyoma is an ill-defined clinicopathological condition that features histologically benign ‘metastatic’ smooth muscle tumor [4]. BML is a very rare disease that has been reported in association with uterine leiomyoma, and about 100 cases have been reported in the literature [5]. The term ‘benign metastasizing leiomyoma’, which was coined initially by Steiner in 1939, is used to describe the presence of benign smooth muscle tumors in an organ distant from the uterus [6]. It affects women of reproductive age who have undergone hysterectomies or myomectomies due to histologically benign-appearing uterine leiomyomas [7,8]. The lung is the most common site of involvement. In addition to pulmonary lesions, lesions of the mediastinum, the nervous system, skin, and bone have been described, with similar growth characteristics [3,9,10]. Although most tumors are asymptomatic and are found incidentally on routine chest X-rays, a few tumors induce cough, dyspnea, and decreased pulmonary function [8,11,12].

## Case presentation

### Case 1

**Figure 1:**
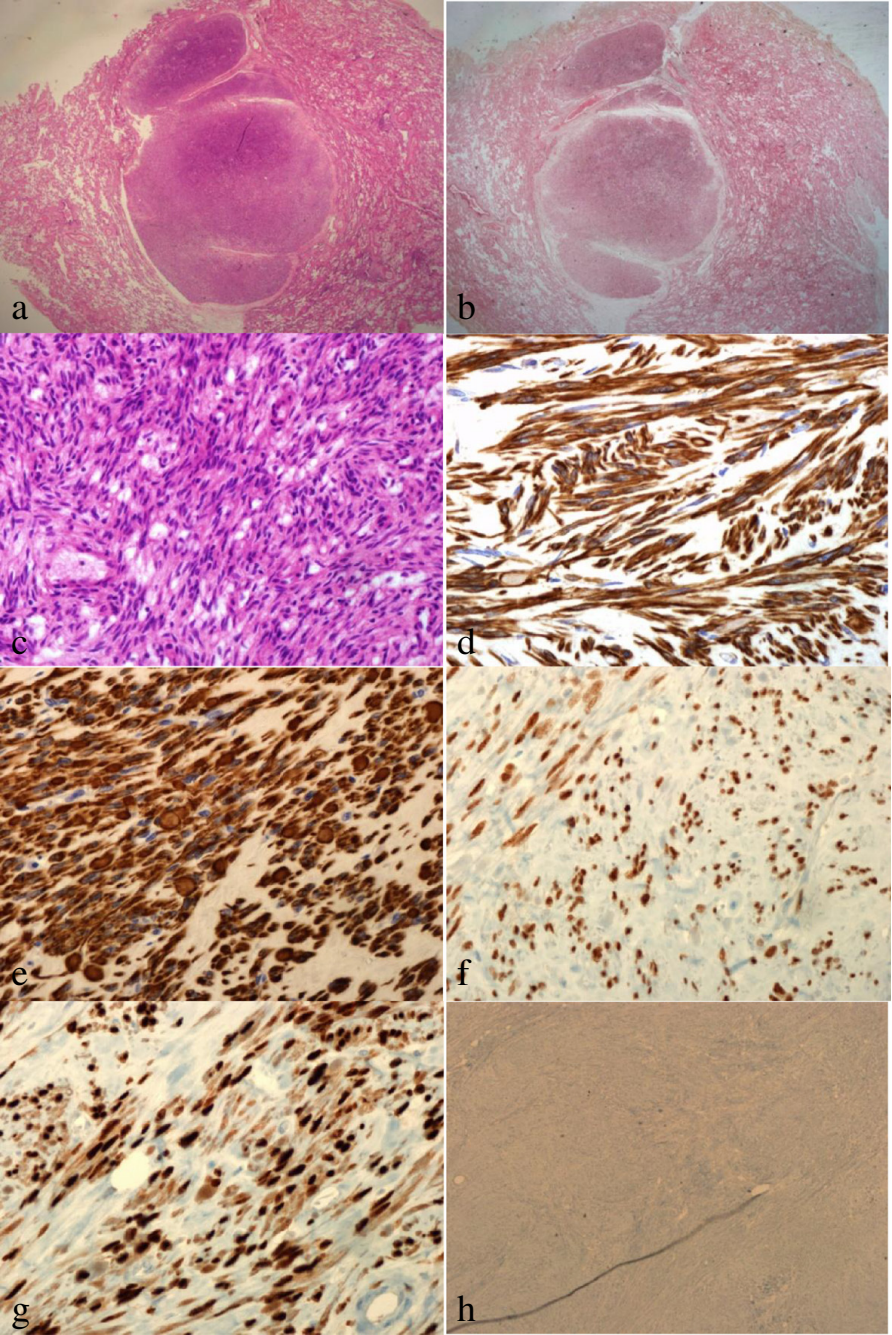
**Histology of the BML. (a)** A well-circumscribed leiomyoma mass in the normal lung parenchyma (H & E, ×12.5). **(b)** The mass is positive for Masson’s trichrome stain. It reveals that the tumor is of smooth muscle nature (Masson’s trichrome, ×12.5). **(c)** Benign-appearing spindle-shaped cells arranged in intersecting fascicles (H & E, ×400). Immunohistochemical staining was positive for: **(d)** smooth muscle actin (×400), **(e)** desmin (×400), **(f)** estrogen receptor (× 400), and **(g)** progesterone (×400). **(h)** Ki-67 is less than 1%, signifying a low proliferate status and a benign nature (×200).

A 53-year-old primiparous woman was referred to the cardiothoracic and vascular surgery outpatient clinic of our hospital for further evaluation of chronic cough and hemoptysis. Chest computed tomography (CT) showed a 10 cm solitary lung nodule with haziness in the left upper lobe. The patient’s breathing sound decreased. She did not have any specific past history. She had entered menopause at the age of 50. She had been diagnosed with a small uterine myoma 3 years prior to this presentation but had not received any treatment. She underwent excisional biopsy of the mass. Pathologic diagnosis was benign metastatic leiomyoma. Masson trichrome staining revealed that the tumor cells were of smooth muscle nature (Figure 1a,b). Immunohistochemical staining was positive for actin. She did not receive further treatment and has remained healthy for 16 years.

### Case 2

**Figure 2:**
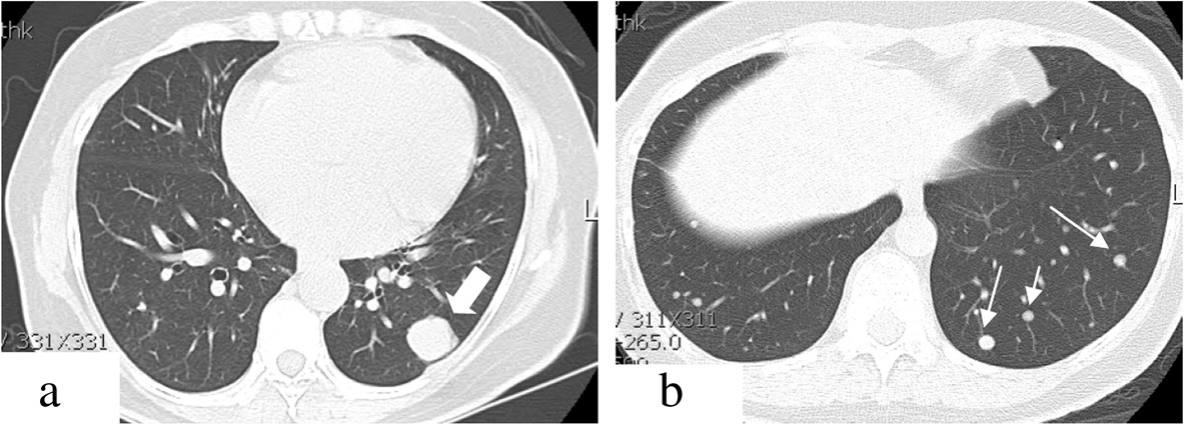
**Chest computed tomography (CT) shows multiple pulmonary nodules. (a)** Largest nodule measures 2.7 × 4.4 cm (thick white arrow). **(b)** Small variable nodules are seen in both lungs (narrow white arrow).

A 50-year-old woman (gravid 3, para 3) presented at our clinic with a chief complaint of menorrhagia. Her menstrual periods had been regular prior to this presentation. Transvaginal ultrasonography showed a 10 × 10 cm myoma that was compressing the endometrium. A chest X-ray showed small multiple nodules of variable size in both lower lung fields. She had not previously had pulmonary disease, such as pulmonary tuberculosis or pneumonia. She did not complain of any chest symptoms, such as cough, dyspnea, or chest pain. Chest CT showed multiple small well-defined scattered pulmonary nodules in both lungs. The largest nodule measured 2.7 × 4.4 cm (Figure 2). The patient underwent a total laparoscopic hysterectomy along with a video-assisted thoracoscopic lung biopsy. Analysis of the uterine and lung lesions revealed leiomyoma and metastatic leiomyoma, respectively. Immunohistochemical staining was positive for actin (Figure 1c,d), desmin, estrogen receptors, and progesterone receptors. We decided to monitor the remaining residual lung lesion: if the size of the remaining lung lesion increases, then bilateral salpingo-oophorectomy with gonadotrophin-releasing hormone agonist (GnRH) therapy will be instituted. She has remained healthy for 3 months without evidence of disease progression.

### Case 3

**Figure 3:**
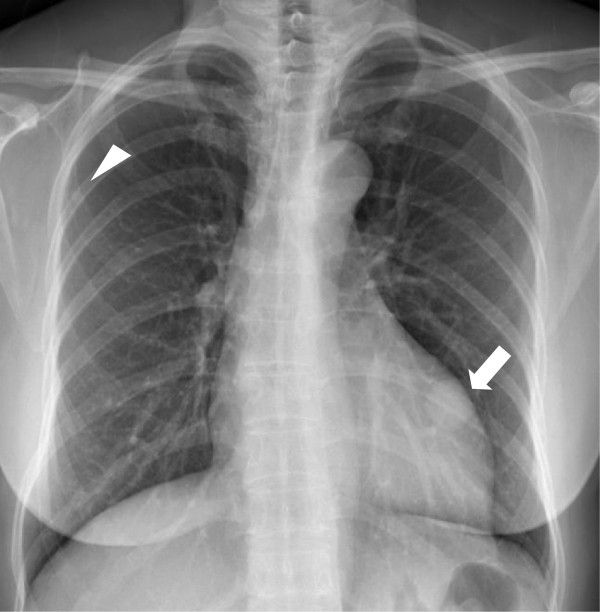
**Chest radiograph.** There is a 2.6-cm well-defined oval mass in the left lower lung (white arrow) and a small ovoid nodule in the right upper lobe field (arrowhead).

**Figure 4:**
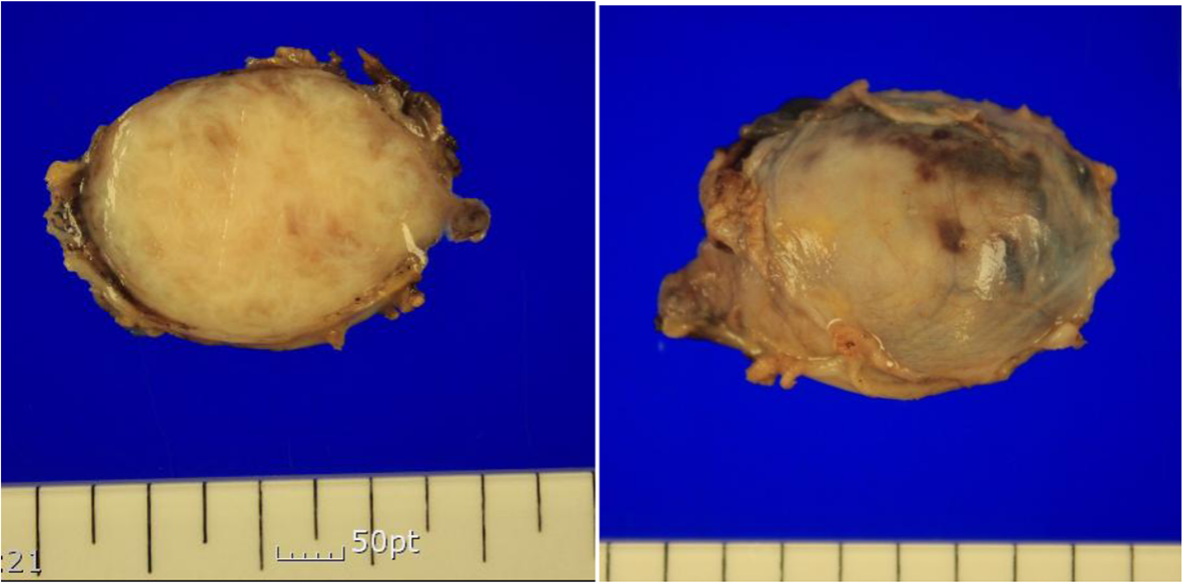
**Photograph of a lung nodule.** An irregular pale brown tissue fragment, measuring 4.0 × 0.5 cm.

A 49-year-old primiparous perimenopausal woman was referred to the cardiothoracic and vascular surgery outpatient clinic of our hospital for further evaluation of incidental lung masses. A chest X-ray showed a 2.5-cm round nodule in the left lower lobe and a smaller well-defined ovoid nodule in the right upper lobe (Figure 3). The patient underwent myomectomy at the age of 42 years and total abdominal hysterectomy at the age of 43 years, due to recurrence. There was no significant family history. The patient had no pulmonary symptoms. Chest CT showed a 2.5-cm well-defined round nodule with poor contrast enhancement in the left lower lobe and a smaller well-defined ovoid nodule in the right upper lobe. The patient underwent a video-assisted thoracoscopic wedge resection of the left lower lobe and right upper lobe.The mass shows a irregular pale brown tissue fragment (Figure 4). Pathologic diagnosis was leiomyoma of the lung. Immunohistochemical staining was positive for desmin and actin (Figure 1e). Transvaginal sonography showed no abnormal findings other than atrophic ovaries. Because the patient was perimenopausal, we decided to monitor her for the progression of the disease. She was healthy without evidence of progression of disease 2 months following the surgery.

### Case 4

A 48-year-old nulliparous perimenopausal woman visited our clinic with a huge abdominal lump. She was diagnosed with systemic lupus erythematosus and underwent uterine myomectomy. She had undergone uterine myomectomy 11 years back. Uterine myoma recurred 1 year ago. Computed tomography showed a 10.5 × 10.5 cm heterogenous, low-density lesion on the right side of the uterus and multiple low-attenuated foci on the left side. There were multiple small nodules in both lungs. The patient underwent total abdominal hysterectomy along with left adnexectomy. She underwent lung needle biopsy of the multiple nodules. Pathologic diagnosis was leiomyoma of the uterus and lung. Immunohistochemical staining was positive for actin ,desmin,estrogen receptor and progesterone receptor (Figure 1f,g) Ki-67 is less than 1% (Figure 4h). We decided to observe the residual lung lesion. The patient was healthy without recurrence, for 2 years following the surgery.

## Discussion

Benign metastasizing leiomyoma is a uterine leiomyoma with pulmonary metastasis occurring in young adulthood, especially during the premenopausal period. Martin [13] classified leiomyomatous lung lesions into three categories in 1982: (1) BML in women, (2) metastatic leiomyoma in men and children, and (3) multiple pulmonary fibroleiomyomatous hamartoma, occurring in any subjects. The author reported that these are all pathologically identical but, on the basis of clinical manifestations, BML and multiple pulmonary fibroleiomyomatous hamartomas are separate disease entities. In this study, the BMLs of the lung in women were hormone-sensitive, so they have good prognoses.

The pathogenesis of BML of the lung has not yet been completely identified. Various pathogenetic mechanisms have been proposed: hormone-sensitive *in situ* proliferation of smooth muscle bundles [14], benign smooth muscle cells transported from a uterine leiomyoma and colonized in the lung, and low-grade uterine leiomyosarcoma metastasized to the lung [15]. The possibility of surgically induced mechanical displacement from the preexisting benign uterine tumor has been suggested because BML usually develops several years after the resection of uterine leiomyomas but rarely after Cesarean section [14,16,17]. Recently, Nucci *et al.*[18] described consistent chromosomal aberrations (19q and 22q terminal deletion) in BML cases and suggested that BML can be affected genetically.

Of our four patients, two who had been diagnosed with BML were also diagnosed with myoma at our clinic. Kayser *et al.*[19] reported that the mean interval between hysterectomy and the development of lung lesions was 14.9 years. In our two cases, the mean interval between hysterectomy and the development of lung lesions was 9 years.

Pathological features are of a benign nature. Cytologic atypia, coagulative tumor cell necrosis, increased mitoses (> 5 per 10 high-power field) with a low Ki-67 index, and the absence of high cellularity, support the low proliferative state and the benign nature of these tumors. Histologic examination reveals interlacing fascicles of smooth muscle cells without anaplasia or vascular invasion, with entrapped respiratory epithelium [5]. Various immunohistochemical markers, such as desmin, muscle-specific actin, and vimentin, confirm mesenchymal derivation with smooth muscle differentiation of these tumors [20]. The presence of estrogen and progesterone receptors suggests the derivation of BML from the female genital tract, which supports the rationale for treatment with hormonal agents [20,21].

Radiologically, BML appears as a well-circumscribed nodule a few millimeters to a few centimeters in size and can be found as a solitary lesion or as multiple lesions scattered within the normal interstitium [22]. It does not enhance with intravenous contrast medium. Endobronchial and pleural sparing is characteristic of BML [5,22]. Military patterns, cavitary nodules, interstitial lung disease, and multiloculated fluid-containing cystic lesions have rarely been reported in BML [12,23-25]. Horstmann *et al.*[26] reported, in a radiologic evaluation, that multiple nodules (87%, bilateral; 70% and unilateral, 17%) with a solitary nodule occurred only in 13% of all patients. Such nodules may remain unchanged, increase or decrease in size, or, rarely, became cystic [4,12]. In our cases, one patient showed a single solitary nodule, one patient showed two solitary nodules, one in each lung, and two patients showed multiple nodules in both lungs.

Benign metastasizing leiomyoma is similar to lymphangioleiomyomatosis (LAM) in the following aspects: proliferation of the smooth muscle cells, location in the lung, hormonal dependence, and HMB-45 immunoreactivity [27]. Lymphangioleiomyomatosis showed proliferation of atypical smooth muscle cells along with lymphatics, blood vessels, and small airways. In contrast, it has been shown that lymphatics, blood vessels, and small airways are spared in BML [28]. Immunohistochemical staining is useful for differential diagnosis between BML and LAM. Human melanoma black (HMB-45) is used to confirm the melanocytic origin of cells. Patients with LAM are positive for HMB-45, while those with BML are negative for HMB-45 [6].

A standard treatment of BML has not yet been established. Because of the hormone-sensitive characteristics of BML, its treatments are based on hormonal manipulation with either surgical or medical oophorectomy. Moreover, regression of metastatic lesions has been demonstrated in situations where estrogen levels fall significantly, especially after termination of pregnancy and menopause [2,10]. For this reason, some authors observed and closely followed up patients after pathologic confirmation in perimenopause or postmenopausal women. The duration of observation differs between reports (6 months to 14 years) (Table 1) [9,29,30]. Hoetzenecker *et al.*[30] proposed a ‘wait-and-see strategy’ and observed one patient with multiple bi-lobar BML for 14 years. Our patients were all in the perimenopausal or postmenopausal state, so we decided to observe them. The follow-up duration ranged from 2 months to 16 years.

**Table 1:** Summary of selected cases of BML

	**Number of patients**	**Interval between uterine surgery and diagnosis of BML**	**Size or type of uterine myoma**	**Adjuvant treatment**	**Outcome (follow-up period)**
Rivera *et al.*[3]	2	11 years	6 cm	GnRH agonist, tamoxifen, aromatase inhibitor	5 years
		3 years	22 × 16 × 10 cm, intramural	GnRH agonist tamoxifen, aromatase inhibitor	2 years
Egberts *et al.*[4].	1	10 years	-	GnRH agonist	3 years
Mogi *et al*. [31].	1	7 years	14 m	GnRH agonist	-
Bodner-Alder *et al.*[32].	1	1 year	6 × 7 cm, subserosal	GnRH agonist	3 months
Nasu *et al.*[10].	1	10 years	-	Aromatase inhibitor	15 months
Beck *et al*. [33].	1	5 years	10 × 10 cm, 8 × 8 cm subserosal	Progesterone	1 year
Wentling *et al*. [34].	1	6 years	5 cm	Progesterone	3 years
Goyle *et al*. [11].	1	5 years	-	progesterone	3 months
Moon *et al*. [9].	1	-	6 × 5 cm	observation	6 months
Awonuga *et al.*[35].	2	3 years	21 cm	Observation	-
		6 years	Six, ~30 cm (multiple)	Tamoxifen	2 years
Ki *et al.* (this case report)	4	None	Small	Observation	16 years
		None	10 × 10 cm	Observation	3 months
		7 years	8 × 8 cm, 5 × 7 cm	Observation	2 months
		11 years	10.5 × 8 cm, subserosal	Observation	2 years

Reversible medical castration with GnRH agonists, which suppress endogenous gonadotropin secretions required for gonadal steroid production, has been described with good therapeutic outcomes in several reports [3,4,31,32]. Egberts *et al.*[4] reported that treatment with GnRH agonists suppressed lung nodules without any increase in size for a period of 36 months. Mogi *et al.*[31] also indicated that the use of GnRH agonists can lead to shrinkage of lung nodules.

Progesterone therapy has been shown to be effective in both prophylaxis against recurrence and regression of BML [11,33,34,36]. The basis for the use of progestin lies in its ability to suppress the hypothalamic-pituitary-gonadal axis, thereby reducing ovarian estrogen synthesis. Moreover, progesterone increases the enzymatic inactivation rate of estradiol and reduces aromatase activity by up to 30% [3]. Wentling *et al.*[34] documented a complete disappearance of lung lesions after treatment with oral progestin, (megestrol acetate) at a dose of 0.04 g three times daily for 3 months, even in the presence of intact ovarian function. Beck *et al.*[33] reported regression of lung lesions with oral progestin after total hysterectomy along with bilateral salpingo-oophorectomy one year after operation.

Estrogen receptor antagonists, such as tamoxifen, are used to treat BML. Säynäjäkangas *et al.*[8] reported a 47-year old woman who had been treated with tamoxifen and had a stable disease for about 1 year. However, Abramson *et al.*[37] reported a BML patient who had been unsuccessfully treated with tamoxifen.

Aromatase inhibitors have been used to treat BML. Aromatase-P450, an enzyme involved in the last step of estrogen biosynthesis, is widely distributed throughout the body. Anastrozole and other selective nonsteroidal inhibitors of this enzyme reduce estradiol concentrations by acting on both the gonads and peripheral and tumor tissues [3]. Nasu *et al.*[10] reported a 46-year-old woman who was treated with oral anastrozole after total hysterectomy with bilateral salpingo-oophorectomy and showed a stable disease for 15 months after operation.

The clinical course of BML varies from a chronic asymptomatic course to a rapid progression leading to respiratory failure and death. Bachman *et al.*[38] analyzed the disease course of 24 BML patients and reported that 13% of the patients died within two years, and 46% survived longer than four years. They also stated that the longest survival period was 36 years (one patient with extensive lung involvement). One of the important prognostic factors is thought to depend on the estrogen or progesterone status of the patient because BML is associated with hormonal receptors [39,40]. Horstmann *et al.*[26] mentioned that the disease process is indolent in postmenopausal patients, while progressive respiratory compromise and even death occur in the premenopausal patients. In this context, Nasu *et al.*[10] documented that an individual treatment strategy should be considered for each patient depending on the size and location of the tumor and the hormone receptor status.

## Conclusions

In this report, we reviewed four cases of perimenopausal or postmenopausal women with BML who were followed up after surgery. BML is a rare disease, and its standard treatment remains to be established. Because the clinical course of BML varies among cases, an individual approach should be considered.

## Consent

Written informed consent was obtained. The study was approved by the Institutional Review Board (KC13ZISE0412).

## Abbreviations

BML: Benign metastasizing leiomyoma; CT: Computed tomography; H & E: Hematoxylin and eosin.

## Competing interests

The authors declare that they have no competing interests.

## Authors’ contributions

EYK wrote the initial draft. SYH and JSP performed the surgery and helped collect clinical information. SY, KHL, and SJH designed the study and wrote the manuscript. All authors have read and approved the final manuscript.
